# The Treatment of Delay in the First Stage of Natural Labor, with Special Reference to the Early Use of the Forceps

**Published:** 1882-05

**Authors:** Joseph Eve Allen

**Affiliations:** Augusta, Ga.


ATLANTA
### MEDICAL REGISTER.

VOL. I.] MAY, 1882. [No. 8

### Original.

### THE TREATMENT OF DELAY IN THE FIRST STAGE OF NATURAL LABOR, WITH SPECIAL REFERENCE TO THE EARLY USE OF THE FORCEPS.

BY JOSEPH EVE ALLEN, M.D., AUGUSTA, GA.

*Read Before the Augusta Academy of Medicine, March 3d*, 1882.

Some obstetric authorities, governed, perhaps, by too strict an adherence to the old maxim, that meddlsome midwifery is bad, have recently held that during the progress of the first stage of labor no harm can result to either mother or child so long as the liquor amnii has not escaped and the membranes are unruptured ; and that hence, no matter for what length of time this state of affairs may be protracted, active interference on the part of the accoucheur is never necessary. The truth of this statement is, however, contradicted by almost every day experience, for cases often occur in which the labor is retarded many hours and the woman brought almost into a condition of exhaustion, because, owing to excessive toughness of the membranes or from a state of hydramnion, the advance of the head is obstructed or efficient uterine contraction prevented. Such cases terminate rapidly and satisfactorily after the tense amniotic sac is artificially broken.

When rightly considered, it will be readily seen that long continuance of the first stage, whether the membranes are ruptured or not, is attended with just as serious if not more hurtful consequences, as undue delay in the second stage. The constant pressure of the child's head upon the soft and delicate tissues of the lower portion of the body of the uterus and cervix produces an irritable, edematous, and congested condition of these parts: the persistence and severity of the pains prevent rest and sleep, break down the woman's strength, and unfit her for muscular exertion during the stage of propulsion; and also pain being in itself the most powerful cerebro spinal depressant known, her vital energies are greatly taxed and her capacity for resisting the dangers of the puerperal period much impaired. Each contraction of the uterus, whether with or without pain, becomes, as Dr. Tyler Smith has shown, after a certain time a distinct shock to the system, and thus exerts a most depressing and exhausting influence; and the uterine muscle, like every other muscle of the body, after long continued ineffectual effort, falls first into a state of tonic or tetanic contraction, and then into a state of relaxation or inertia. In the first condition there is liability to rupture of the womb, or asphyxia of the child from arrest of the placental circulation; and the second greatly predisposes to the occurrence of post partum hemorrhage.

The causes of delay in the first stage of natural labor that may require the aid of the obstetrician to complete the delivery, are rigidity of the os uteri, either spastic or organic, premature rupture of the membranes, want of proper relation and adaptation of the head to the cervix, inefficient and irregular action of the uterus; or strong contractions with no progress, owing to pelvic deformity, large ossified head or large shoulders, short cord, or to the cord being wrapped several times around the child.*

When unfavorable symptoms occur during the first

---

*Shortness of the Cord as a Cause of Obstruction in Natural Labor. By J. Mathews Duncan, M.D., Amer. Jour. Obts., February, 1882.

stage, or difficulties are foreseen that will make the subsequent delivery unnatural, farther delay is dangerous, and assistance necessary; there are two courses of practice open to pursue, the choice of which is to be determined by the case in hand, viz: either to arrest the labor for a time by the administration of some form of opium, or to hasten the delivery by effecting dilation of the os by means of digital manipulation, the use of Barnes bags, the forceps, the knife, etc.

In the majority of cases, especially in those in which the cervix is unobliterated and the os internum but little dilated, much good can be accomplished by the administration of a full dose of opium. Dr. Wm. T. Lusk* says on this subject: "Opiates often accomplish wonders in one of two ways: when, owing to the prolongation of the labor and its attendant pain, the patient's nervous energies have become exhausted, the arrest of pain enables the woman to sleep, and, with the recuperation of power that comes upon awakening, good pains follow, which bring the labor to a happy termination. In other cases, after the employment of the anodyne the parts apparently relax, and an acceleration of the labor follows. In these cases the oxytocic effect is probably due to the quieting action exerted upon the spinal nerves. It has been surmised that the nerves of the uterus derived from the cerebro-spinal system possess inhibitory properties, a theory, which, if true, readily explains how severe pain suspends uterine action, and how the quieting of pain would restore to the motor nerves their full energy." When an opiate is indicated McMun's elixir of opium in teaspoonful doses is the preparation that best serves the purpose, for the reason that it is equal to laudanum in its narcotic effect, and does not produce constipation or nausea.

The routine practice of giving in every case of labor hydrate of chloral during the first stage, has been attended in a great many instances with the good result of preventing the occurence of uterine inertia and exhaustion, and alleviating in a marked degree the sufferings of the woman, and thus obviating the necessity for instrumental or other

*Science and Art of Midwifery, page 424, interference. This drug is hypnotic and not narcotic in its effect, and hence it obtunds the sensibility to pain without stopping the action of the uterus, it calms nervous excitement, lengthens the interval between the pains, and thus increases the power and efficiency of the uterine contractions. To produce this result fifteen grains of hydrate of chloral must be taken every quarter of an hour until sleep is produced.

Analgesics are of no benefit in certain cases of protracted first stage, because either their action is of too short duration, lasting only from ten to thirty minutes, or after they have exerted their effect upon the system, the same abnormal condition still remains which, at first, caused the delay; therefore, in such cases, it is necessary to make efforts to hasten the delivery.

Dilatation of the os can often be accomplished by the finger: this should only be attempted, however, when the cervix is thin, soft and relaxed, or, in other words, in the condition known as that of dilatability, and when practiced should always be done between or during the subsidence of the pains, for the reason that the contraction of the muscular fibres of the uterus starts at the crevix and passes upward to the fundus, and hence, the os always becomes firmer and smaller at the commencement and dilates toward the end of a uterine contraction. Endeavors to produce dilatation at the beginning will only increase the intensity of the pains, with no other than the bad result of tearing or bruising the muscular tissue. When the crevix is unobliterated and undilated, the hydrostatic dilators of Dr. Robt. Barnes, of London, are a most valuable resource. These bags in overcoming the resistance of the parts by keeping up a firm, equitable, graduated pressure, closely imitate the process of nature: for, normally, the opening of the mouth of the womb is an entirely passive process, similar to that of sphincters of the bladder and bowels—a yielding under pressure from above—and is effected, not as some have supposed, by the contraction of the longitudinal muscular fibres pulling up the cervix; but by the amniotic sack, and presenting part being pressed into it by the contraction of the fundus.*

*Barnes Obstetric Operations, page 102.

The application of belladonna ointment, or the use of the hot douche against the cervix as a means of promoting relaxation, is generally of but little value, and not to be relied on.

The use of such powerful oxytocics as ergot or cotton-root is of course not to be considered before complete dilatation of the os, except in cases of placenta prævia or accidental hemorrhage; for these drugs, by causing tetantic spasm of the womb, might produce rupture of that organ, or death of the child from the constant pressure exerted upon it. The use of bi-sulphate of quinia at this time has however been advised by Dr. Fordyce Barker, of New York, and Dr. Albert H. Smith, of Philadelphia, and the good results which they have obtained, makes this drug worthy of a more extended trial by the profession. The bi-sulphate of of quinia is to be given in one large dose of fifteen grains, and it is claimed that it thus acts as an excitant and stimulant to the hypertrophied muscular fibres of the parturient uterus, producing strong, regular and perfect contractions. The known influence of quinia in contracting the unstripped fibres of the middle coat of the blood vessels upon which depends its great value in preventing congestion and inflammation* sustains this theory. A more probable explanation of the beneficial action of this drug during the first stage of labor has, however, been given by Dr. H. F. Campbell,† who states that during labor, incessantly recurring pain without corresponding uterine contractions is due to abnormal reflex excitability of the spinal centres, and that quinia, by acting as a nervous sedative, allays this condition. Quinia, owing to its power in controlling the inflammatory processes, and being as the investigations of Polli and Binz have proved, one of the most useful of internal antiseptics, finds its most appropriate and important place in the therapeutics of the puerperal state.

Inhalation of chloroform sometimes increases the action

---

*Quinine and its modus operandi by A. Sibley Campbell, M.D., Trans. Med. Association of Georgia, 1880.

†Quinine in Gynecic and Obstetric Practice by H. F. Campbell, M.D., Trans. Amer. Gynecological Society, Vol. V.

of the womb by abolishing the inhibitory effect of severe pain, but should never be carried during the first stage of labor to the extent of producing anæsthesia, because this condition is often too transient to be of any benefit and is apt to be followed, as Dr. W. T. Lusk* has shown, by a condition of atony and relaxation, which may make the use of the forceps necessary during the second stage, and which especially favors the occurrence of post partum hemorrhage.

The knife is never to be used to cause dilatation of the cervix except in cases of stenosis or occlusion of the os from the presence of cicatricial tissue or malignant disease. A very interesting case of complete occlusion of the os, in which it was necessary to perform vaginal hysterotomy before dilatation could be commenced, and delivery accomplished, is reported in the Transactions of the American Gynecological Society for 1880, by Dr. Jos. A. Eve, of Augusta, Ga.

When the os does not dilate because the head does not become, as it were, properly fixed against it during a pain, this end can sometimes be attained by directing the woman to hold her breath and bear down, thus reinforcing the uterine contractions by the action of the auxiliary muscles of the abdomen. Bearing down during the first stage, being contrary to the advice of almost all obstetric authors, is to be practised of course with the utmost caution and only in cases of strong and vigorous women, and never in those who are weak and delicate. When, owing to obliquity of the uterus or pendulous abdomen, delay is occasioned by the head not entering the pelvis properly, the use of a stout abdominal binder is often of great service.

The early use of the forceps in the first stage of natural labor was first suggested and practised by Dr. Isaac E. Taylor, of New York, in 1863. This was a decided innovation in practice, for according to the teachings of the older writers and of those who guided the professional opinions of the day, the use of the forceps was to be confined exclusively to the second stage. Thus Dr. Denman† states that

*The Use of Chloroform during Labor, By Wm. T. Lusk, M.D. Trans. Amer. Gynecological Society, Vol. II.

†An Introduction to the Practice of Midwifery, page 371.

"before the completion of the first stage of labor, that is, before the os is perfectly dilated and the membranes broken, the use of the long forceps cannot properly come under contemplation." Dr. Ramsbotham* says, in his directions for the use of the long forceps, that this instrument should never be applied if one-third of the cervix can be felt continuously. Dr. Meigs advises that before the forceps be employed the cervix shall have gone up up entirely over the head of the child and an ear be felt.

Although the progress of scientific knowledge has now greatly changed and modified the views formerly entertained by obstetricians on this subject, still the idea of the use of the forceps, as advocated by Dr. Taylor, through a small undilated os uteri as a means of producing dilatation and expediting the termination of the first stage, has not yet received that full recognition and general acceptance which its great importance demands. Thus Dr. Leishman† says, "complete dilatation of the os is, indeed, in a sense, absolutely essential; and it is certain that a greater degree of dilatation is necessary for this (the use of the forceps) than for any other of the operations for delivery. But complete dilatation, in the sense which would attach to the term, does not imply that the anterior lip of the os has passed out of reach beyond the head, but merely such dilatation as will admit of the safe passage of the head." Schroeder‡ states that the cervix uteri must be obliterated, and the os so far dilated that the head can easily pass through it before the forceps should be applied. Playfair|| says, "If the os be not fully dilated, but is sufficiently so to admit of the passage of the forceps, the operation, under urgent circumstances, may be quite justifiable, although it must necessarily be a somewhat anxious one." Barnes§ says, "When the forceps will pass—and it is quite possible to apply it when the os will allow three fingers to pass as far as the knuckles—this instrument may serve to dilate

*Principles and Practice of Obstetric Medicine and Surgery, page 202.

†System of Midwifery, page 474.

‡Manual of Midwifery, page 174.

||System of Midwifery, page 430.

§Obstetric Operations, page 110.

further. But this must be done with great caution. The head being grasped, you may draw steadily down ; and, by keeping up gentle traction, the wedge formed by the blades and the head will gradually dilate the os, perhaps enough to allow it to pass and thus save the child's life."

Dr. Taylor* advises that neither traction, compression, or leverage, as practised in the second stage, be made by the forceps during the first stage of labor, but that they be used simply to hold the head properly in contact with the internal os during a pain, and then to aid the uterine contractions in effecting obliteration of the cervix and complete dilatation. In some cases this instrument also produces flexion, and often by its presence within the uterus, through reflex action, excites that organ to more regular and perfect contraction. This dynamic power of the forceps is best exerted when the patient is not in a state of anæsthesia, and hence, whenever possible, this operation is to be performed without chloroform. The contact of the cervix should not be constant, but at the commencement of a pain the occiput should be brought down, and held during its continuance and for a short time after it has subsided, and then be allowed to recede in the intervals between the contractions. These manipulations should always be done with the utmost care and patience, and hence cases often require two or three hours to terminate in this manner the first stage.

It is to be especially understood that it is the *early* and not the *frequent* use of the forceps in the first stage of labor that Dr. Taylor advocates, for he states that the cases in which they should be employed occur but seldom, and that they are to be applied only when it is evident that other means cannot accomplish the delivery. On this subject, Dr. Geo. Johnston†, of Dublin, remarks : "This practice should never be adopted merely in order to save our own time, nor should it be attempted when the os uteri is rigid ; in such cases the proper means should be taken to

---

*The Early Use of the Forceps in the First Stage of Natural Labor. By Isaac E. Taylor, M.D , Trans. Amer. Gynecological Society, Vol. IV,

†Report of the Rotunda Lying-in Hospital for the Year 1875. Obst. Jour. Great Britain and Ireland, May, 1876.

overcome this state, and it should alone be had recourse to when the life of the mother or that of her offspring is in jeopardy."

The forceps that Dr. Taylor has devised for use early during the first stage of labor are chiefly peculiar in having very narrow blades, so that it is possible to introduce them through an os measuring from an inch and a half to seven-eighths of an inch in diameter, or even as small as a nickel piece. The lock is a modification of the French lock, to which a spring is attached, by means of which a slight amount of compression is made, sufficient to cause the instrument to be self-retaining.

Clinical experience has proved that it is erroneous to suppose that there is any especial danger in the introduction of the forceps within the uterine cavity, or that the danger is proportional to the smallness of the os at the time of the operation; for often when applied during the second stage the blades are necessarily passed high up into the interior of the womb without any attendant bad results; and Dr. Taylor has shown there is no more harm done by the proper use of the forceps during the first stage, through an undilated os, than during the second stage through a small contracted vulval orifice. For by ocular examination of many living patients, as well as by careful investigation of post mortem specimens, he has proved that lacerations of the cervix in such cases very seldom occur. Dr. George Johnston,* of Dublin, from statistics of one hundred and sixty-nine cases under his charge as Master of the Rotunda Lying-in Hospital, in which the forceps were applied before the os uteri was fully dilated, concludes that "the practice is not alone safe and justifiable, but also a great preservative of the lives of both mothers and children."

As the practicability, safety, and life-saving usefulness of the operation has been conclusively demonstated, time will certainly establish the early use of the forceps, in certain cases of delay during the first stage of labor, as one of the most valuable and conservative of modern advances in operative midwifery.

*Trans, Obstetrical Soc. of Dublin, Obstetrical Jour. Great Britain and Ireland, March, 1879.

